# Co-occurrence of iron, folate, and vitamin A deficiency among pregnant women in eastern Ethiopia: a community-based study

**DOI:** 10.1186/s40795-023-00724-x

**Published:** 2023-06-23

**Authors:** Meseret Belete Fite, Abera Kenay Tura, Tesfaye Assebe Yadeta, Lemessa Oljira, Tara Wilfong, Newas Yusuf Mamme, Gemechu Asefa, Demiraw Bikila Gurmu, Wossene Habtu, Feyissa Challa Waka, Nahom Tefera Demiss, Meseret Woldeyohannes, Masresha Tessema, Dawit Alemayehu, Tahir Ahmed Hassen, Aboma Motuma, Kedir Teji Roba

**Affiliations:** 1 Department of Public Health, Institute of Health Sciences, Wollega University, Nekemte, Ethiopia; 2 School of Nursing and Midwifery, College of Health and Medical Sciences, Haramaya University, Harar, Ethiopia; 3 Department of Obstetrics and Gynaecology, University Medical Centre Groningen, University of Groningen, Groningen, the Netherlands; 4 School of Public Health, College of Health and Medical Sciences, Haramaya University, Harar, Ethiopia; 5 Department of National Clinical Chemistry Reference Laboratory, Ethiopian Public Health Institute, Addis Ababa, Ethiopia; 6 Food Science and Nutrition Research Directorate, Ethiopian Public Health Institute, Addis Ababa, Ethiopia

**Keywords:** Co-occurrence, Micronutrient deficiency, Pregnant women, Ferritin, Retinol, Vitamin A, Ethiopia

## Abstract

**Background:**

It is well known that the magnitude of undernutrition in Ethiopia is unacceptably high. The burden of co-occurrence of iron, folate, and vitamin A deficiency, on the other hand, has received less attention. Thus, in this study, we looked at the prevalence of iron, folate, and vitamin A deficiency in pregnant women in eastern Ethiopia.

**Methods:**

A community-based cross-sectional study was conducted among 397 pregnant women in Haramaya district, eastern Ethiopia. An interview-assisted questionnaire and blood serum were collected from pregnant women using standard techniques and shipped to an EPHI for micronutrient analysis. Factors associated with the co-occurrence of iron, folate, and vitamin A deficiency were identified using binary and multiple logistic regressions.

**Results:**

According to this study, 81.6% of the participants were deficient in at least one micronutrient, and 53.53.2% were deficient in two or more. Women who did not receive iron-folic acid supplementation (AOR = 2.44; 95% CI = 1.52–3.92), did not attend Antenatal care (ANC) follow up (AOR = 2.88; 95% CI = 1.81–4.61), and reported low consumption of diversified diet (AOR = 2.18 (95% CI = 1.35–3.51) had a higher risk of co-occurrence of iron, folate, and vitamin A deficiency.

**Conclusion:**

This study found that more than half of pregnant women were in multiple micronutrients, indicating a major public health issue. In addition to the IFA supplementation programs that are already in place, there is a need for multiple micronutrient supplementation.

## Introduction

Micronutrients constitute minerals, vitamins, and trace elements that are required in small amounts for biological functions, called the ‘magic wands’ of health by the World Health Organization (WHO) [1]. Micronutrients support the body to yield enzymes, hormones, and other substances that are vital for proper growth and development. Although the body requires a small quantity of micronutrients, the impacts of their lack are severe [2]. Micronutrients play important biological roles during the reproductive years and are vital in preparing a woman for pregnancy [3, 4]. Although micronutrient deficiency affects all age groups, pregnant women tend to be one of the most at-risk groups [5]. As such, deficiencies during pregnancy are related to several adverse outcomes for the mother and her newborn: prenatal anemia, maternal and perinatal death, low birth weight, preterm birth, intra-uterine growth restriction, altered immune response, and cognitive deficits in the baby [5–8].

Micronutrient deficiencies in pregnancy are common in low resource settings like Ethiopia. A recent systematic review revealed that 35.6% of pregnant women in Sub-Saharan Africa [9] and 35.6% in Ethiopia [10] were anemic. Folate deficiency is yet a severe public health problem, particularly among pregnant women in developing countries [11, 12]. It has been associated to several complications in gestation [13], and frequently considered as a potential risk factor for happening neural tube defects (NTD) in the fetus; affecting more than 300,000 children globally, and 65 babies out of 10,000 births in Ethiopia [12, 14]. Vitamin A deficiency (VAD) remains the leading cause of preventable blindness and serous a public health issue in developing countries [15]. Many investigations related vitamin A deficiency during pregnancy with several adverse pregnancy and birth outcomes [16]. The existing previous studies [17, 18] carried out in the Ethiopia indicted higher prevalence figures ranging from 17 to 37.9%.

According to World Health Organization (WHO), the most common micronutrient deficiencies are iron, vitamin A and iodine deficiencies. These are followed by zinc, folic acid (vitamin B9), vitamin B12 and other B-group vitamins, vitamin C, vitamin D, calcium, selenium, and fluoride [19]. Although supplementation of iron ad folic acid has become part of routine care during antenatal care (ANC), providers’ compliance in providing counseling and provision of the tables seems inadequate [20]. In addition, women’s compliance in taking the prescribed tablets as required is limited. For example, the 2016 Ethiopian Demographic and Health Survey (EDHS) study found that only 5% of pregnant women took IFA supplements for at least 90 days [21]. For individual nutrients such as ferritin, vitamin A, or folate, evidence on the level of micronutrient deficiency, its effect on pregnancy outcome, and factors associated with such deficiencies is well established [18, 22, 23]. To the best of our knowledge, no research has been conducted on the co-occurrence of iron, folate, and vitamin A deficiency among pregnant women in Ethiopia, particularly in eastern Ethiopia. In this study, we assessed the prevalence of iron, folate, and vitamin A deficiency in pregnant women in eastern Ethiopia.

## Methods

### Study settings and period

The study was embedded into the Haramaya Health Demographic Surveillance and Health Research Centre (HDS-HRC) established in 2018 by Haramaya University. HDS-HRC is established to be a comprehensive and sustainable data source for monitoring population health and demographic events in the Haramaya district. Profile of HDS-HRC and other details are described elsewhere [24]. The detail description of this study has been given elsewhere in the previous paper [25, 26]. This study was conducted from January 5 to February 12, 2021.

#### Study design and population

This study is part of a larger study on pregnant women's nutritional status in HDS-HRC, which is also described elsewhere [26]. In brief, a cross-sectional community-based study was conducted among randomly selected pregnant women who lived in specific kebeles for at least six months during the study period. Women with a recent history of anemia or on anemia treatment, as well as those with chronic illnesses (acute or chronic liver disease, heart disease, chronic renal failure, diabetes mellitus, hypertension, etc.), gestational diabetes, pregnancy-induced hypertension, and acute or chronic blood loss) were excluded from the study because their health does not reflect that of the general population.

#### Sample size and sampling procedures

The sample size was calculated using a single population proportion formula with the following assumptions: a 50% prevalence of at least two micronutrient deficiencies with a 95% confidence interval and a 5% margin of error. After accounting for a 10% non-response rate, the final sample size calculated was 422. However, because this study was part of a larger longitudinal study (a prospective cohort study aimed at assessing neonatal birth weight and its association with maternal iron status), the same 475 pregnant women were included. Previous papers published detailed sampling methods and procedures [25, 26].

#### Data collection

Face-to-face interviews, anthropometric measurements, and blood collection were conducted by trained research assistants. Data on socioeconomic, obstetric, maternal perception, food consumption, dietary diversity, knowledge, attitude, and practices of pregnant women were collected through face to face interviews. This methods of data collections were published elsewhere [21, 27–32]. Then mid-upper arm circumference (MUAC) and maternal height measurement were followed the interviews. The formerly validated food frequency questionnaire (FFQ), dietary diversity scores (DDS), The food variety score (FVS) and food consumption score and Household Food Insecurity Access Scale (HFIAS) were collected and analyzed [27–32]. A 5 ml venous blood sample was drawn by trained and experienced laboratory professionals for serum analysis of ferritin, folate, retinol concentration, and serum high-sensitive C-reactive protein (hsCRP). In addition to this women dietary diversity were calculated [31]. A detailed description of data collection can be found in previous papers [25, 26].

#### Biochemical measurement

An experienced laboratory technologist used a serum separator tube (SST) to collect whole blood (5 ml) for 30 min. Samples were temporarily stored at -80'C until analysis, at which point they were transferred to the Ethiopian Public Health Institute (EPHI) for examination. At the EPHI National Reference Laboratory, we measured serum hsCRP using an immune-turbidimetric assay (reagent CRPHS Ref. 04628918190), ferritin using the Sandwich electrochemiluminescence principle (reagent ferritin Ref. 03737551190), and folate using the Competition electrochemiluminescence principle (reagent folate Ref. 07559992190). Serum retinol levels were also measured.

#### Data quality assurance

Data collectors, laboratory professionals, and supervisors were trained for two days, and the questionnaire was pre-tested on 5% of pregnant women in a nearby district, with adjustments made based on the results. Supervisors closely monitored data collection, double-checking it daily before entry. The national laboratory analysis was performed at the EPHI, which is internationally accredited (accreditation no. M 0025).

### Data processing and analysis

Epi-data 3.1 was used to double-enter the data, and Stata 14 was used to clean and analyze it. The outcome variable (concurrent micronutrient status) was dichotomized as deficient if at least two of the three micronutrients were deficient (coded as 1), or no if none of the three micronutrients were deficient (coded as 0). (coded as 0). The factors associated with micronutrient deficiency were then identified using a binary logistic regression. After adjusting for multicollinearity, all binary regression variables with p 0.25 were added to multiple logistic regression models. Serum iron, retinol, and folate deficiency, as well as serum CRP levels, were measured and classified in accordance with their respective standards [33–35].

The principal component analysis was used to calculate the wealth index (PCA). The index was calculated using 41 household variables, including ownership of latrines, agricultural land and size, selected household assets, livestock quantities, and source of drinking water. Nutritional knowledge and attitudes toward iron-rich diet consumption were assessed using a Likert scale and the PCA; factor scores were totaled and classified into tertiles.

### Ethical considerations

This study was conducted in agreement with the Declaration of Helsinki-Ethical principle for medical research involving human subjects. The proposal was approved by the Institutional Health Research Ethics Review Committee (IHRERC) of the College of Health and Medical Sciences, Haramaya University (ref No: IHRERC/223/2020). Written informed consent was obtained from all participants and legally authorized representatives "of minors below 16 years of age and illiterates” and confidentiality was maintained by excluding all personal identifiers.

## Results

### Socio-demographic characteristics

**Fig. 1:**
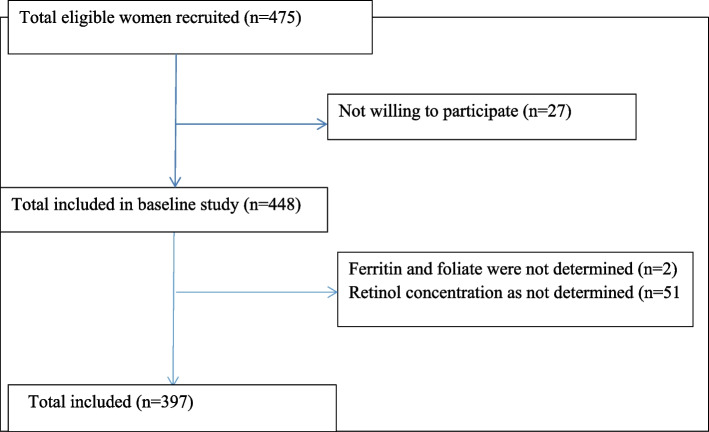
Flowchart of the study

In this study, 397 women were included, for whom all biochemical test results were available (Fig. 1).

The majority of respondents (73.05%) could not read or write, were housewives (96.73%), farmers (93.45%), and had a family size of 1–5 (76.32%). Furthermore, only 21.66% of respondents were in the richest quintiles (Table 1).

**Table 1:** Socio-demographic characteristics of pregnant women in Haramaya District, Eastern Ethiopia, 2021 (*n* = 397)

Variables	Frequency(*n* = 397)	Percentage (%)
**Age (years) Mean (±SD)**	**24.97(±5.15)**	
< 18	21	5.29
18–35	353	88.92
> 35	23	5.79
**Educational level of the woman**		
Can’t read or write	290	73.05
Read or write	24	6.05
Formal education	83	20.91
**Educational Level of husband**		
Can’t read or write	217	54.66
Read or write	54	13.62
Grade 1–8	102	25.69
Grade 9 and above	24	6.05
Occupation of the woman		
Housewives	384	96.73
Merchants	13	3.27
**Occupation of husband**		
Farmers	391	93.45
Daily labors	26	6.55
**Family size**		
1–5	303	76.32
> 5	94	23.68
**Agricultural land possession**		
No	246	61.96
Yes	151	38.04
**Wealth Index (Quintile)**		
Poorest	80	20.1
Poor	77	19.40
Middle	73	18.39
Rich	81	20.40
Richest	86	21.66
**Parity**		
0(primiparas)	91	22.92
1–4	262	65.99
≥5	44	11.08

### Level of Factors associated withco-occurrence of iron, folate, and vitamin A deficiency

This study found that 53.15% (95% CI: 48, 58) of people had more than two deficiencies, while 16% had three. Table 2 shows the individual and co-occurrence of iron, folate, and vitamin A deficiency (Table 2).

**Table 2:** Micronutrient deficiency among pregnant women in Haramaya district, eastern Ethiopia, 2021 (*n* = 397)

Variables	Frequency (*n* = 397)	Percentage (%)
Ferritin deficiency	212	53.40
Folate deficiency	198	49.87
Vitamin A deficiency	191	48.11
Combination of micronutrient deficiency		
Ferritin and folate deficiencies	140	35.26
Ferritin and vitamin A efficiencies	110	27.71
Folate and vitamin A deficiencies	93	23.43
No micronutrient deficiency	73	18.39
One micronutrient deficiency	324	81.61
Two micronutrient deficiencies	145	36.52
Three micronutrient deficiencies	66	16.62
(2 ≥) micronutrient deficiency	211	53.15

### Factors associated withco-occurrence of iron, folate, and vitamin A deficiency

In the bivariable analysis, age, women's educational level, family size, ANC follow up, IFA supplementation, source of drinking water, knowledge of micronutrient rich foods, restriction of the intake of some foods and dietary diversity, and khat chewing were found to be candidate variables for multivariable analysis at, *p* < 0.25.

In the final multivariable logistic regression model, after adjusting for potential confounders, ANC follow up, IFA supplementation, and dietary diversity were found to be the independent predictors of the **co-**occurrence of iron, folate, and vitamin A deficiency. The risk of co-occurrence of iron, folate, and vitamin A deficiency was 2.44 times more likely among women who didn’t receive IFA supplementation (AOR = 2.44; 95% CI = 1.52–3.92), 2.88 times more likely among who didn’t attend ANC follow up (AOR = 2.88; 95% CI = 1.81–4.61,) and 2.18 time more likely among those who reported low consumption of diversified diet (AOR = 2.18 (95% CI = 1.35–3.51) compared to their counterparts, respectively (Table 3).

**Table 3:** Factors associated with concurrent micronutrient deficiency among pregnant women Haramaya district, Eastern Ethiopia 2021

Variables	**co-occurrence of iron, folate, and vitamin A deficiency**		COR (95%CI)	AOR (95%CI)	*P*-value
	Yes (*n* = 211)	No (*n* = 186)			
Age of the women in a years					0.158
< 18	8 (3.79)	13(6.99)	1	1	
18–35	200 (94.79)	166(89.25)	1.96 (0.79,4.84)	2.07 (0.75,5.68)	
> 35	3 (1.42)	7(3.76)	0.74 (0.14,3.50)	0.85 (0.14,5.10)	
Women’s educational level					0.845
Illiterate	174 (82.46)	140 (75.27)	1	1	
Literate	37 (17.54)	46 (24.73)	0.65 (0.40,105)	0.95 (0.54,1.65)	
Family sizes					0.199
1–5	156 (73.93)	149 (80.11)	1	1	
> 5	155 (26.07)	37 (19.89)	0.42 (0.88,2.28)	1.40 (0.84,2.36)	
ANC follow up					0.001*
Yes	55 (26.07)	83 (44.62)	1	1	
No	156 (156)	103 (55.38)	1.67(1.07,2.60	2.88(1.8,4.61)	
IFA supplementation					< 0.001**
Yes	54 (25.59)	137 (73.66)	1	1	
No	89 (42.18)	49 (26.34)	2.15 (1.40,3.28)	2.44 (1.52,3.92)	
Source of drinking water					0.408
Unprotected	86 (40.76)	95 (51.08)	1	1	
Protected	125 (59.24)	91 (48.92)	1.52 (1.019,2.26)	1.22 (0.76,1.98)	
Knowledge of micronutrient**-**rich foods					0.211
No	196 (92.89)	164 (88.17)	1	1	
Yes	15 (7.11)	22 (11.83)	0.57 (0.28,1.14)	0.62 (0 29,1.31)	
Khat chewing					0.111
No	72 (34.12)	82 (44.09)	1	1	
Yes	139 (65.88)	104 (55.91)	1.52 (1.01,2.28)	1.43 (0.92,2.23)	
Restriction of the intake of some foods					0.999
No	148 (70.14)	114 (61.29)	1	1	
Yes	63 (29.86)	72 (38.71)	0.74 (0.44,1.02)	0.67 (0.41,1.80)	
Dietary diversity					0.001*
High	48 (22.75)	72 (38.71)	1	1	
Low	163 (77.25)	114 (61.29)	1.69(1.06,2.69)	2.88(1.8,4.61)	

**Statistically significant at *p*- value < 0.001; * statistically significant at *p*-value < 0.05

## Discussion

The prevalence of concurrent micronutrient deficiency was 53.15% (95% CI: 48, 58) in this study. Co-occurrence of iron, folate, and vitamin A deficiency was more common in women who did not take regular IFA supplementation, but less common in women who received antenatal care and reported adequate dietary diversity.

The co-occurrence of more than one or two micronutrients among pregnant women in low and middle-income countries is well documented [36, 37]. The present study reveals that pregnant women in rural Haramaya district are likely at risk of multiple micronutrients deficiencies. Our finding is lower than the finding of a study carried out in Nepal [38] which reported 82% prevalence of coexistence of two or more micronutrient deficiencies. However, the result of the current study is lower than the finding of the study that documented in Ghana [39]. The possible reason for the variation might be due to the differences in the number of micronutrients measured in which the studies.

In the current study the individual micronutrient (iron, folate and Vitamin A) deficiencies were comparable probably resulting in part from a shared nutrient insufficiency of good dietary sources such as meat, inadequate of which is consumed in this area [25]. Another reason might be a combined adverse effect of no regular consumption of micronutrient-rich foods and diet that is usually high in inhibitors of mineral absorption [40].

Micro-nutrient nutrient intakes are often low among women in sub Saharan Africa [41], including Ethiopia [42] because access to micronutrient-rich foods and fortified foods is limited, and these foods are expensive, locally unavailable, or unacceptable for cultural or religious reasons. Moreover, the reason for the higher prevalence could result of poor quality diets and amplified physiological requirements, which are intensified by inadequate health systems, poverty and inequities, and by socio-cultural aspects (early marriage, and traditional food habits) [43]. Concomitant nutritional deficiencies could decrease the likely benefit of a specific nutrient supplement in improving nutrition status and morbidity [44]. Antenatal multiple micronutrient supplement use has emerged as an important public health intervention for women in low-income countries and has benefits for pregnancy outcomes over and above IFA, which is currently recommended for pregnant women by the WHO [45].

We discovered that eating a diverse diet during pregnancy is significantly associated with a lower risk of iron, folate, and vitamin A deficiency co-occurring. This is consistent with previous research conducted in African countries [39, 46, 47]. The possible explanation is that women with a more diverse diet consume significantly more meat, legumes, and nuts. Furthermore, the vegetables and fruits consumed in the groups with the highest dietary diversity are high in vitamin C, which increases iron absorption. As a result, it is possible to conclude that consuming a diverse diet may affect serum ferritin concentration by changing the intake of diet items to a higher consumption of iron-rich diet. Furthermore, proper food consumption may result in a link between food intake from different food groups and adequacy of micronutrient intake. As a result, public health awareness must be emphasized in order to encourage a higher intake of various food groups during pregnancy.

The current study discovered that antenatal care is associated with a lower risk of having multiple micronutrient deficiencies, which is consistent with research conducted in developing countries [39, 48, 49]. This could be because antenatal care offers unique opportunities for nutrition communication and provides insights into women's experiences obtaining nutrition information during pregnancy [50]. Thus, dietary counseling could improve nutrient, vitamin, and mineral intake from food and supplementation during pregnancy. This implies that achieving adequate micronutrient consumption during pregnancy will necessitate strengthening the delivery and utilization of maternal nutrition services integrated into ANC services in the health system. Therefore, because pregnant women need more support to optimize food and nutrient intakes, making sure that women get quality ANC services and access to nutrition information is really important.

Furthermore, regular IFA consumption was linked to a lower risk of iron, folate, and vitamin A deficiency, which is consistent with previous research [51, 52]. This could be because the supplementation is specifically designed for the two micronutrients (iron, and folate). As a result, it is suggested that women receive regular IFA to ensure positive outcomes for both mothers and their children, which will affect future generations.

The fact that the study was the first to assess the level of concurrent micronutrient deficiency among pregnant women in Ethiopia was its main strength. In addition, an analysis of conceptually important confounder (CRP) was performed and was used for serum ferritin concentration adjustments in the current study to make a decision on the co-occurrence of inflammation in the interpretation of iron status findings. The findings of this study should also be interpreted with some caution. Due to limited resources, this study looks at parameters for assessing iron, folate, and vitamin A deficiencies in pregnancy, which may underestimate the magnitude of concurrent micronutrient deficiency.

Based on this study. We recommend promoting the intake of a diversified diet, making sure that pregnant women get quality ANC to ensure good outcomes for both mother and her child that affect the next generation are very important. While the influence of deficiency of individual micronutrients—ferritin, folate, and vitamin A—on pregnancy outcomes is well established, there is a need to understand how this concomitant malnutrition affects women and offspring. There is a need towards multiple micronutrients supplementation in addition to the started folic acid supplementation programs. Thus, antenatal multiple micro-nutrient supplement use has emerged as an important public health intervention for women in low-income countries and has benefits for pregnancy outcomes over and above IFA is documented, there is a need to understand the effectiveness of these supplements in improving the micronutrient status of pregnant women in the Ethiopian context, where concurrent micronutrient deficiency is prevalent.

## Conclusion

More than half of pregnant women in eastern Ethiopia were found to be iron, folate, and vitamin A deficient. The independent predictors of the deficiency were dietary diversity, ANC follow-up, and IFA supplementation. This demonstrates that micronutrient deficiencies are a significant public health issue in Ethiopia.

## Data Availability

All data are available within the manuscript. Additional data can be obtained from the corresponding author on a reasonable request.

## Abbreviations

ANC: Antenatal care
ASF: Animal source foods
DDS: Dietary diversity score
EDHS: Ethiopian Demographic and Health Survey
IDA: Iron deficiency anemia
IFA: Iron and folic acid
HDS-HRC: Haramaya Demographic Surveillance and Health Research Center
MN: Micronutrient
MMN: Multiple Micronutrient
CA: Principal Component Analysis
WHO: World Health Organization
